# Brief reviews

**DOI:** 10.1590/S1980-57642011DN05030012

**Published:** 2011

**Authors:** Sonia Maria Dozzi Brucki

**Affiliations:** 1 Behavioral and Cognitive Neurology Unit, Department of Neurology, and Cognitive Disorders Reference Center (CEREDIC). Hospital das Clínicas of the University of São Paulo School of Medicine, São Paulo SP, Brazil.

## COGNITIVE ACTIVITIES DURING ADULTHOOD ARE MORE IMPORTANT THAN EDUCATION IN BUILDING RESERVE.

**Reed et al. J Int Neuropsychol Soc 2011;17:615-624.**

Cognitive reserve can be defined as a mechanism through which effects of brain pathology can be modified by education, occupation, life style, leisure time activities, and intellectual activities throughout the life course.

Authors analyzed four determinants for cognitive reserve: education, cognitively stimulating leisure time activities at two timepoints in life (at the age of 40 and at baseline), and socioeconomic status (SES). Neuropathological measures were also analyzed in two community-based longitudinal studies of cognitive aging; the Religious Order Study (ROS) and the Rush Memory and Aging Project (MAP); involving a total of 652 autopsied cases. All had been evaluated at baseline and annually up to death.

The total variance explained by neuropathology for individual cognitive domains was as follows: episodic memory, 34.9%; working memory, 32.3%; visuospatial ability, 31.2%; perceptual speed, 35.1%.

Cognitive activity at age 40 and in later life, at the time of the baseline evaluation, had stronger relationships with reserve whereas cognitive activity at age 40 had the strongest relationship, independent of the contribution of cognitive activity at baseline evaluation.

Results suggest that leisure cognitive activities (reading, writing letters, keeping a diary, visiting the library, and attending concerts) throughout adulthood are more important than education in determining cognitive reserve.

## THE ALZHEIMER’S ASSOCIATION EXTERNAL QUALITY CONTROL PROGRAM FOR CEREBROSPINAL FLUID BIOMARKERS.

**Mattsson et al. Alzheimer’s Disease & Dementia 2011;7:386-305.**

CSF biomarkers for AD can be monitored and measured: amyloid β-42 (Aβ-42), total-tau (T-tau), and phosphorylated-tau (P-tau). They can be detected before dementia onset and have high diagnostic accuracy for AD. However, these biomarkers levels differ among studies with different accuracies being reported.

During the International Conference on AD in Vienna, it was decided to initiate an international quality control (QC) program for AD CSF biomarkers. The program consists of: a standardized operating procedure for lumbar puncture and CSF sample handling procedures, and an external comparison program of CSF analyses among laboratories.

This report discloses data from the Alzheimer’s Association QC program for AD CSF biomarkers. The total coefficients of variation among laboratories ranged from 13% to 36%. Going forward with the QC program, the most likely causes for the variations can be identified and addressed. It is very important to verify the underlying causes for this variability in order to decrease disparities among laboratories and attain homogeneous accuracy.

## CLINICOPATHOLOGICAL CORRELATIONS IN CORTICOBASAL DEGENERATION.

**Lee et al. Ann Neurol 2011;70:327-340.**

This report verifies cognitive and behavioral features, physical findings, and brain atrophy patterns in pathology-proven corticobasal degeneration (CBD) and corticobasal syndrome (CBS) with known pathology. It reports on 18 patients with autopsy-proven CBD and 40 patients with CBS as first presentation with known pathology.

The clinical notes were reviewed and the dominant clinical syndrome at first presentation was determined, dividing cases into: behavioral variant FTD (bvFTD), progressive nonfluent aphasia (PNFA), posterior cortical atrophy (PCA), and executive motor phenotype (EM).

In the CBD cohort, 16 patients had pure CBD pathology. Behavioral or cognitive problems were the initial symptoms in 15 of the 18 patients; a movement disorder was present at onset in only 4 out of 18 patients. Compared to controls, CBD patients showed atrophy in dorsal prefrontal and perirolandic cortex, striatum, and brainstem.

Autopsies for patients with CBS evidenced: 9 AD, 14 CBD, 5 PSP, 5 FTLD-TDP. Patients with CBS-AD were younger and had higher functional impairment at first evaluation. CBS was associated with posteromedial frontal and perirolandic cortex and dorsal insula atrophy.

There was no difference in survival among groups. Initial symptoms for CBD-PNFA patients involved language difficulties, followed by motor symptoms 1 to 5 years later; EM-CBD patients presented with a variety of motor symptoms, in some patients there was coincident onset of cognitive or behavioral changes; most common symptoms in the bvFTD-CBD cases were social withdrawal and motor symptoms; and one patient with PCA-DCB presented with difficulty reading, and trouble using their right hand 2 years later.

This study shows us the wide spectrum of CBD. CBD is the most common pathological substrate in CBS patients, but occurs in less than half of patients.

## DEMENTIA IN PARKINSON’S DISEASE: A 20-YEAR NEUROPSYCHOLOGICAL STUDY (SYDNEY MULTICENTRE STUDY).

**Reid et al. J Neurol Neurosurg Psychiatr 2011;82:1033-1037.**

Dementia is a common condition which manifests during the course of PD. The Sydney Multicentre Study of PD recruited patients newly diagnosed as having PD from 1984 to 1987. Clinical and neuropsychological assessments were performed at study entry before treatment initiation (baseline) and at follow-up examinations at 3, 5, 10, 15 and 20 years. Dementia was diagnosed if patient showed cognitive impairment in memory and in two other areas of cognition (<2 SD from the mean cognitive score of the control group).

PD patients with dementia at 5-10 years after diagnosis were classified as having mid-stage PD dementia (MPDD), and those with dementia manifesting 10 years after PD diagnosis were classified as late-stage PD dementia (LPDD).

There were no differences between groups in relation to: age at onset of dementia (70±5.5 y) or age at death (77±5.5 y). A younger age at PD onset was associated with a significantly longer dementia-free survival. PD patients with lower linguistic abilities are likely to have cognitive impairment earlier in their disease.

## LACK OF EVIDENCE FOR THE EFFICACY OF MEMANTINE IN MILD ALZHEIMER DISEASE.

**Schneider et al. Arch Neurol 2011;68:991-998.**

Memantine is indicated for treatment of moderate to severe AD patients. Authors analyzed all clinical trials of memantine versus placebo that included patients with mild AD.

The total number of patients with mild AD was 431, representing 38.2% of the total number of AD patients (n=1128, who completed the trials).

There was no evidence for the efficacy of memantine on any of the outcomes in mild AD patients. Similarly, no significant effects were evident on the ADAS-cog (cognitive measure), the CIBIC-plus (clinical impression), the ADCS-ADL scale (functional measure), or the NPI – Neuropsychiatric Inventory (behavioral measure).

In patients with moderate AD, a small difference was detected on the ADAS-cog and the CIBIC-plus, but no differences were observed on the ADCS-ADL scale or the NPI.

